# A systematic dissection of sequence elements determining β-Klotho and FGF interaction and signaling

**DOI:** 10.1038/s41598-018-29396-5

**Published:** 2018-07-23

**Authors:** Sally Yu Shi, Ya-Wen Lu, Jason Richardson, Xiaoshan Min, Jennifer Weiszmann, William G. Richards, Zhulun Wang, Zhongqi Zhang, Jun Zhang, Yang Li

**Affiliations:** 1 Department of Cardiometabolic Disorders, Amgen Discovery Research, Amgen Inc., 1120 Veterans Blvd., South San Francisco, CA 94080 USA; 2 Department of Attribute Sciences, Amgen Inc., One Amgen Center Drive, Thousand Oaks, CA 91320 USA; 3 Department of Therapeutic Discovery, Amgen Discovery Research, Amgen Inc., 1120 Veterans Blvd., South San Francisco, CA 94080 USA

## Abstract

Endocrine fibroblast growth factors (FGFs) require Klotho transmembrane proteins as necessary co-receptors to activate FGF receptor (FGFR) signaling. In particular, FGF19 and FGF21 function through β-Klotho to regulate glucose and lipid metabolism. Recent research has focused on elucidating how these two FGFs interact with β-Klotho and FGFRs to activate downstream signaling. In this study, using hydrogen deuterium exchange coupled to mass spectrometry (HDX-MS), we identified regions on the β-Klotho protein that likely participate in ligand interaction, and vice versa. Alanine and arginine mutagenesis were carried out to further probe the contributions of individual residues to receptor/ligand interactions. Using biochemical and cell-based signaling assays with full-length proteins, we show that both the KL1 and KL2 domains of β-Klotho participate in ligand interaction, and these binding sites on β-Klotho are shared by FGF19 and FGF21. In addition, we show that two highly conserved regions in the C-terminal tail of FGF19 and FGF21 are responsible for interaction with the co-receptor. Our results are consistent with recent publications on the crystal structures of the Klotho proteins and provide insight into how endocrine FGFs interact with co-receptors for signal transduction.

## Introduction

Fibroblast growth factors (FGFs) are a group of structurally-related, secreted signaling molecules that regulate diverse cellular functions including cell survival, growth, differentiation and migration^1^. Paracrine FGFs show high affinity towards the extracellular matrix (ECM) component heparan sulfate (HS), and are thus retained in the ECM and function locally. In contrast, endocrine FGFs, including FGF15/19, FGF21 and FGF23, have reduced affinity for HS^2^ and can therefore escape from the ECM into the circulation to reach their targets in distant organs^3–5^. All FGF proteins share a conserved globular core domain consisting of 12 antiparallel β-strands arranged into a β-trefoil structure^6^. The N- and C-terminal regions that flank the conserved core domain are highly varied in primary sequence and length^6^.

FGFs signal through FGF receptors (FGFRs) which are single-pass transmembrane proteins with three extracellular immunoglobin-like (D1–D3) domains and an intracellular tyrosine kinase domain^6^. Paracrine FGFs utilize HS as a cofactor for high affinity interaction with FGFRs^7,8^. Binding of FGF ligand induces FGFR dimerization and tyrosine kinase activity, leading to activation of downstream signaling^9^. Endocrine FGFs, on the other hand, have intrinsically poor affinity for their cognate FGFRs and require two transmembrane proteins, α-Klotho and β-Klotho, as necessary co-receptors for signaling^2,10^.

Klotho proteins consist of an extracellular domain (ECD), a single-pass transmembrane region and a short cytoplasmic tail^11,12^. The ECD contains two tandem repeats (termed KL1 and KL2) that share sequence similarity with family 1 glucosidases^13^ but have recently been shown to lack intrinsic enzymatic activity^14,15^. It is believed that these two co-receptors serve primarily as a docking site or scaffold to facilitate the interaction of endocrine FGFs with FGFRs^16^. Alpha-Klotho serves as the co-receptor for FGF23^16,17^, and β-Klotho is the co-receptor for both FGF19 and FGF21^18–21^. FGF19 and FGF21 effectively improve metabolic parameters in diabetic rodent models^22–28^, and as such, the associated metabolic benefits have promoted considerable interest in therapeutic development against this signaling pathway^29–37^.

Multiple structures of paracrine FGFs, FGFRs, and FGF/FGFR complexes have been resolved over the years^4^. These structural studies mapped detailed interactions between FGFs and FGFRs and provided molecular insights into paracrine FGF functions. For endocrine FGFs, some features of the interactions have been characterized through biochemical approaches. For example, endocrine FGFs have been shown to engage Klotho co-receptors via their C-terminal tails, while the N-terminus is proposed to mediate signal activation^38–41^. Using mutant or chimeric FGFR proteins, the D3 domain of FGFR1c was shown to be involved in co-receptor binding and determination of receptor specificity for FGF21^42,43^. Nonetheless, detailed structural mechanism underlying the interaction of Klotho co-receptors with cognate ligands remained elusive until recently. The slow progress in structural elucidation was in part due to difficulties in producing high-quality recombinant proteins and being able to form stable complexes with multiple components *in vitro*. Recent breakthroughs have revealed the crystal structure of the ligand-bound β-Klotho ECD, as well as a trimeric complex structure composed of α-Klotho ECD, the ligand-binding domain of FGFR1c, together with FGF23^14,15^. While these structures greatly enhanced our understanding of receptor interaction and signaling of the endocrine FGFs, the ligand-bound β-Klotho ECD structure only contained FGF21’s C-terminal domain, and FGF19 co-receptor interactions remain to be fully characterized.

In order to further understand and define the interaction of FGF19 and FGF21 with receptor complexes, we used hydrogen deuterium exchange (HDX) coupled to mass spectrometry (MS) followed by site-directed mutagenesis. Our results show that surfaces on both the KL1 and KL2 domains of β-Klotho participate in interactions with two conserved, distinct sites in the C-terminal tails of FGF19 and FGF21. Thus, through systematic dissection of binding elements between β-Klotho and its ligands at the amino acid level, we propose a model for the assembly of the ligand/receptor complexes.

## Results

### Identification of FGF21-binding surface on β-Klotho

**Figure 1:**
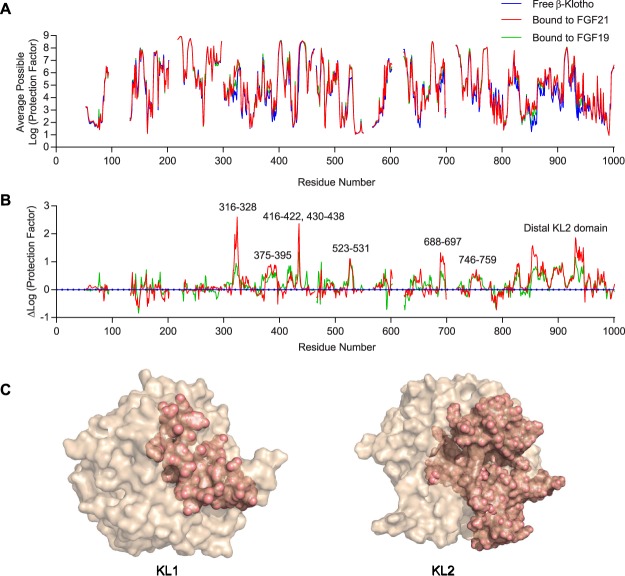
Identification of potential FGF19 and FGF21 interaction regions on β-Klotho by HDX-MS. (**A**) Average possible protection factor plot and (**B**) differential protection factor plot of ligand-bound and free β-Klotho. The blank regions are residues not covered by the data. (**C**) Illustration of potential FGF21 interacting regions (salmon) identified by HDX-MS.

To determine the binding interface between β-Klotho and its cognate ligands FGF19 and FGF21, we employed HDX-MS, which measures isotopic exchange of hydrogen in protein backbone amides. Amides buried in the interface with binding partners will be protected against hydrogen-deuterium exchange, and sequence-specific measurements using MS reveal the location of these protected regions, making HDX-MS a sensitive method for probing protein-protein interactions^44,45^. Figure 1A shows the average possible protection factor of each backbone amide hydrogen in the ECD of the β-Klotho molecule, in both free and ligand-bound forms. The overall protection profiles of β-Klotho were very similar between free and bound forms, indicating that FGF19 or FGF21 binding did not significantly change the overall folding of β-Klotho, which is consistent with recent structural data showing no significant change to the structure of either KL domain upon binding of the FGF21 C-terminal peptide^14^.

To identify β-Klotho residues that likely participate in ligand interaction, we computed the difference in log (protection factor) for each residue between the free and ligand-bound states of β-Klotho. The resulting differential protection factor plot reveals the additional protection conferred by ligand binding, reflected by positive and easily identifiable peaks (Fig. 1B). From the differential protection factor plot, we determined that residues potentially involved in FGF21 binding included residues 316–328 (α5 helix), 375–395 (β5-α6 loop), 416–422 (β7 strand and β7-α7 loop), 430–438 (α7 helix), 523–531 (β9 strand), 688–697 (β11 strand and β11-α11 loop), 746–759 (β12-α12 loop) and the membrane proximal region of the KL2 domain. The differential protection factor plot of β-Klotho upon binding to FGF19 was very similar to FGF21, except that the effects were weaker (Fig. 1B). These regions were then mapped onto a structure of KL1 and a model of KL2 constructed using KL1 as a template^46^. As shown in Fig. 1C, while the protected regions spread discontinuously throughout the linear β-Klotho ECD sequence, when mapped onto the KL1 structure and KL2 model, all regions with a positive peak by HDX-MS clustered on the same surface on KL1 and KL2.

Due to the low, peptide-level resolution of the HDX-MS technology and the fact that ligand binding often stabilizes a greater area than the actual interaction site, residues exhibiting increased protection after ligand binding may encompass an area greater than the actual residues directly involved in interaction. To confirm the HDX-MS mapping results and to obtain a higher resolution mapping of the binding site, we performed scanning mutagenesis to analyze putative residues individually. We first determined accessible surface amino acids from the structures of KL1 and KL2 (Table 1). All residues identified by HDX with a solvent-accessible area greater than 10 Å^2^ were then individually mutated to alanine and arginine. The DNA sequence encoding the ECD of human β-Klotho fused to 6 × His tag at the C-terminus was used as the template for site-directed mutagenesis PCR. Mutant constructs were transiently expressed in Chinese Hamster Ovary (CHO)-EBNA1 suspension cells and conditioned media were collected to profile β-Klotho expression and ligand binding characteristics.

**Table 1:** Surface exposed residues that have a solvent accessible surface area > 10 Å^2^ in positive peaks identified by HDX-MS.

Residue	Residue No.	Surface area (A^2^)	Residue	Residue No.	Surface area (A^2^)	Residue	Residue No.	Surface area (A^2^)
F	317	88.8	T	530	35.4	R	861	171.9
K	318	72.3	E	531	78.6	L	862	197.9
Q	321	61.4	L	662	88.5	A	863	58.3
K	380	107.7	A	750	71.7	R	877	72
P	381	121.5	N	751	112.9	D	893	75.8
A	386	41.4	P	752	101.8	D	894	32.5
K	387	120.8	Y	753	248.3	Q	895	165.7
M	388	57.4	A	754	27.7	A	896	36.2
G	389	58.7	D	755	68.7	L	897	73.5
N	391	68.5	S	756	79.3	E	898	58.5
V	392	62.1	H	757	32.3	D	899	131.5
L	394	48	W	758	49.2	R	901	183.1
N	395	29.1	R	759	125.6	L	902	88.8
F	420	29.8	R	787	84.2	K	904	40.3
D	422	56.7	T	828	21.1	Y	905	107.1
T	430	51.7	I	847	24.8	Y	906	66.9
T	431	76.7	Q	848	167.9	G	908	13.1
Y	434	165.7	L	850	158.2	K	909	65.8
M	435	27.6	D	852	67.9	K	926	54.2
K	437	87.9	I	853	24.8	E	936	40.3
C	523	87.7	R	855	131.9	K	937	92.1
S	526	49.8	S	857	44.4	S	938	139.7
W	527	191.1	S	858	28.9	K	939	79.4
G	528	35.5	P	859	93	R	941	37.4

Western blot analyses were performed to ensure all mutants were expressed at the expected size in CHO-EBNA1 cells (data not shown) and an ELISA assay was used to quantify β-Klotho expression levels in culture media. As shown in Supplementary Fig. S1, expression levels varied between different β-Klotho mutants; seven of the mutants were not detected. It is possible that these seven residues are crucial for proper protein folding or that the mutation of these residues impairs detection by a conformation-sensitive antibody used in the ELISA assay. In our downstream experiments, only conditioned media from β-Klotho mutants having an expression level greater than 0.3 µg/mL were used.

**Figure 2:**
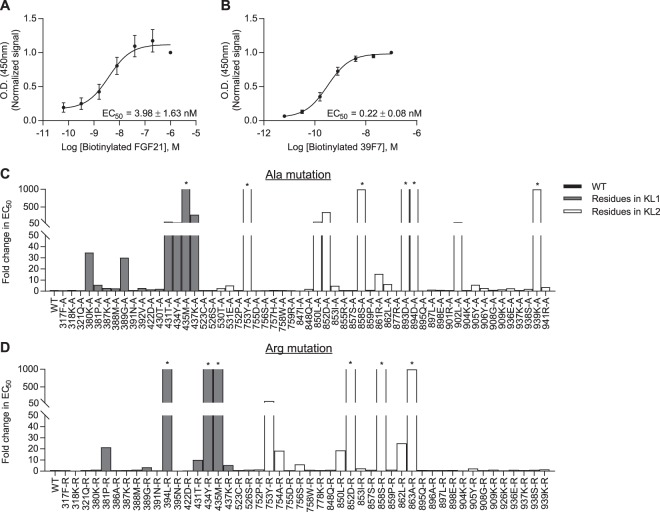
Identification of FGF21 interacting sites on β-Klotho by solid-phase binding assay. (**A**,**B**) Binding of β-Klotho in WT CM to (**A**) FGF21 and (**B**) 39F7, an anti-β-Klotho antibody, measured by solid-phase binding assay. Results are normalized to the response at the highest concentration of the biotinylated proteins. The curves and EC_50_ values are mean ± SD of at least 3 independent experiments performed in duplicates. (**C**,**D**) EC_50_ values determined from the solid-phase binding assay between β-Klotho and FGF21 for (**C)** alanine and (**D**) arginine mutants. EC_50_ values are expressed as fold change relative to WT CM. *Binding curve did not converge.

Next, to study the effects of β-Klotho mutants on ligand interaction, we developed a solid-phase assay whereby β-Klotho is captured from conditioned media and its ability to bind FGF21 measured (Fig. 2A). Using this interaction assay format, the affinity of wild-type (WT) β-Klotho for FGF21 was determined to be in the low nanomolar range (EC_50_ = 3.98 ± 1.62 nM). We also assessed the interaction of WT and mutant β-Klotho to an anti-β-Klotho antibody, 39F7, as an independent control to verify that the folding of these β-Klotho mutants were not significantly affected by the mutations (Fig. 2B and Supplementary Fig. S2). Interaction with the anti-β-Klotho antibody was largely unaffected by single amino acid substitutions (Supplementary Fig. S2), suggesting that the overall structure of the β-Klotho mutants was maintained, and therefore, any observed changes in FGF21 binding likely resulted from the direct participation of these mutated residues or that the mutations secondarily altered the interaction surface on β-Klotho. As shown in Fig. 2C and D, most residues did not appear to be involved in ligand interaction as the mutations, compared to WT, did not lead to significant changes in EC_50_ values. For residues 434Y, 435M, 753Y, 850L, 852D and 858S, replacement by alanine and arginine resulted in significantly attenuated binding to FGF21. This data suggests that two distinct binding sites, one on each of the β-Klotho homologous KL domains, mediate interaction with FGF21, and close contact of both sites with FGF21 is likely required for stable complex formation.

**Figure 3:**
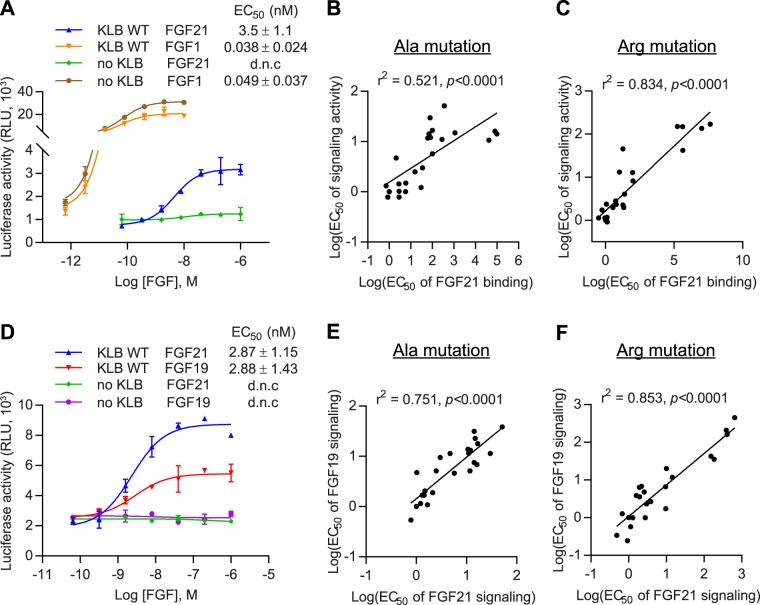
Interaction with β-Klotho determines signaling activity of FGF21. (**A**) Stimulation of Elk1-luciferase reporter activity by FGF1 and FGF21 in HEK293T reporter cells transiently transfected with full-length WT β-Klotho constructs. (**B**,**C**) Linear regression analysis of EC_50_ of FGF21 binding versus EC_50_ of signaling activity for β-Klotho (**B**) alanine and (**C**) arginine mutants. (**D**) Stimulation of Elk1-luciferase reporter activity by FGF19 and FGF21 in HEK293T reporter cells. (**E,F**) Linear regression analysis of EC_50_ of FGF19 signaling activity versus EC_50_ of FGF21 signaling activity for β-Klotho (**E**) alanine and (**F**) arginine mutants. For (**A**) and (**D**), the curves are representative of three independent experiments performed in duplicates. EC_50_ values represent mean ± SD of three independent experiments.

Next, we addressed whether reduced binding to FGF21 affected the ability of the β-Klotho mutants to mediate FGFR activation. To measure FGF21 signaling, we used an FGF21-responsive human embryonic kidney (HEK) 293T reporter cell line which expresses FGFR endogenously (Supplementary Fig. S3). The same set of alanine and arginine mutants were generated on the human full-length β-Klotho expression construct, and mutant constructs were transiently transfected into the reporter cells. All mutants were expressed at a similar level as assessed by SDS-PAGE and immunoblotting of cell lysates (data not shown). After overnight starvation, cells were stimulated with FGF21 and luciferase activity was measured as a readout for β-Klotho/FGFR complex activation. As expected, FGF1, a prototypical paracrine FGF, activated FGFR signaling irrespective of β-Klotho expression level (Fig. 3A; orange and brown curves). On the other hand, FGF21 only induced luciferase activity in the presence of β-Klotho, and its potency was significantly lower than that of FGF1 (Fig. 3A; blue and orange curves). We then profiled the full-length β-Klotho mutants for their ability to mediate FGF21 signaling. As shown in Fig. 3B and C, the EC_50_ values obtained from the reporter assay and from the solid-phase binding assay for both alanine and arginine mutants showed a positive correlation, suggesting that weakened interaction between FGF21 and β-Klotho is directly associated with diminished FGF21 functional activity. Together, this data is consistent with previous reports^18–21^ and provides additional cell-based evidence supporting β-Klotho as an obligatory co-receptor for FGF21 signaling.

We also assessed the functional impact of β-Klotho mutations on FGF19 signaling. In the same reporter system, FGF19 exhibited similar potency but lower maximum response compared to FGF21 in activating Elk1 activity (Fig. 3D; red versus blue curve). Treating β-Klotho-transfected cells with FGF19 showed that the same mutants that affected FGF21 signaling also had an impact on FGF19 functional activity, with a strong correlation between the EC_50_ values of the two assays (Fig. 3E and F). These results are consistent with our HDX-MS data and indicate that FGF19 and 21 share similar binding sites on β-Klotho.

### Identification of β-Klotho-binding sites on FGF21

**Figure 4:**
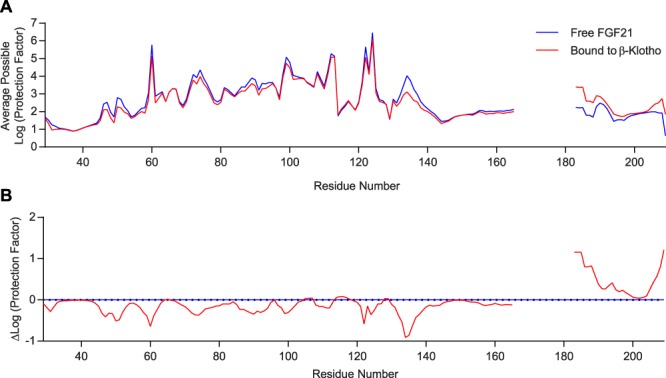
Identification of potential β-Klotho interaction regions on FGF21 by HDX-MS. (**A**) Average possible protection factor plot and (**B**) differential protection factor plot of β-Klotho-bound and free FGF21. The blank regions are residues not covered by the data.

**Figure 5:**
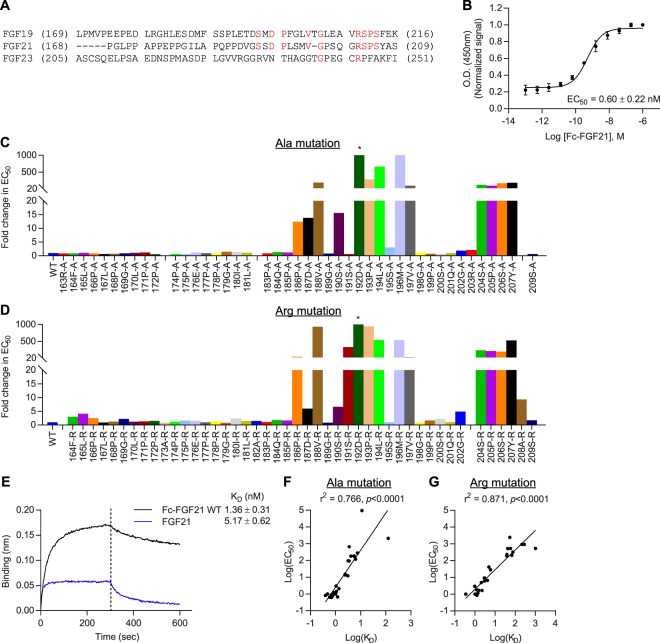
Two distinct sites in C-terminal region of FGF21 interact with β-Klotho. (**A**) Amino acid sequence alignment of C-terminal regions of human FGF19, FGF21 and FGF23. Residues that are identical between FGF19, FGF21 and FGF23 are colored red. (**B**) Binding of WT Fc-FGF21 to soluble human β-Klotho measured by solid-phase binding assay. Results are normalized to the response at the highest concentration of Fc-FGF21 protein. The curve represents mean ± SD of three independent experiments performed in duplicates. (**C**,**D**) EC_50_ values determined from the solid-phase binding assay of Fc-FGF21 (**C**) alanine and (**D**) arginine mutants. Values are expressed as fold change relative to WT protein. Gaps were inserted to align the two graphs. *Curve did not converge. (**E**) Binding of FGF21 and WT Fc-FGF21 to human β-Klotho measured by bio-layer interferometry. K_D_ values were estimated using a 1:1 binding model. (**F**,**G**) Linear regression analysis of K_D_ determined by bio-layer interferometry versus EC_50_ determined by solid-phase binding assay for Fc-FGF21 (**F**) alanine and (**G**) arginine mutants. Values represent mean ± SD of three independent experiments.

We next sought to identify the co-receptor binding sites on FGF19 and FGF21 using the same approach. For FGF21, due to the peptide-level resolution of the HDX-MS technology, we were not able to obtain a readout of the region covering residues 166–182 (Fig. 4A and B). Consistent with previous reports^38–40^, the differential protection factor plot of FGF21 in free versus β-Klotho-bound forms shows positive peaks in the C-terminal tail of the protein (Fig. 4B), suggesting that β-Klotho binds predominantly to C-terminal residues of FGF21. However, what was not predicted from the previous studies^38,40^ was that two main peaks were visible in the differential protection factor plot, one spanning the region from residues 183 through 198 and the other one in the distal C-terminus from residue 204 to 209. Alignment of the C-terminal sequences of FGF19 and FGF21 shows significant sequence similarity in these two regions, whereas the C-terminal tail of FGF23, which does not bind β-Klotho, is more divergent from these conserved residues (Fig. 5A). This suggests that these two regions may serve as the primary binding sites for β-Klotho. For FGF19, a potential binding site is not discernable from the data, likely due to a low binding affinity under these experimental conditions that failed to produce enough changes in protection from isotopic exchange to be observed (Supplementary Fig. S4).

Similar to β-Klotho, we performed scanning mutagenesis on FGF21 and individually mutated residues 163–209 to alanine and arginine. This region spans the entire C-terminal tail and includes 5 additional residues at the distal end of the β-trefoil core domain. The DNA sequence encoding the mature human FGF21 polypeptide fused with the human Fc fragment at the N-terminus (herein referred to as Fc-FGF21) was used as the template for site-directed mutagenesis PCR. Fc-FGF21 has been shown to retain the β-Klotho-binding affinity and the *in vitro* and *in vivo* activity of native FGF21^32,47,48^. Mutants were transiently expressed in HEK293-EBNA1 cells and purified by protein A affinity chromatography.

Affinity of Fc-FGF21 mutants to β-Klotho was measured by a solid-phase binding assay (Fig. 5B). Single residue mutations in the two C-terminal regions identified by positive peaks in HDX resulted in reduced binding to β-Klotho, as evidenced by a right shift in binding curves and an increase in EC_50_ values compared to WT (Fig. 5C and D). In particular, mutations to 192D, 193P, 194L, 196M in the first region, and the four residues near the distal C-terminal tail in the second region almost completely abolished β-Klotho binding, with more than a 100-fold increase in EC_50_ compared to WT. This data suggests that the affected side chains may directly interact with β-Klotho or may be involved in stabilizing the conformation of FGF21. Importantly, these results indicate that both regions in FGF21 C-terminal tail are involved in mediating a stable interaction with β-Klotho.

Overall, changes in EC_50_ values were consistent between alanine and arginine mutants, except at residue 191S. The alanine mutant did not exhibit significant changes in potency, while replacing the serine with the positively charged arginine attenuated β-Klotho binding, suggesting that the serine side chain do not participate directly in co-receptor interaction but may be in close proximity to the binding interface.

We further analyzed the binding kinetics of Fc-FGF21 mutants by bio-layer interferometry. WT Fc-FGF21 exhibited a 4-fold increase in affinity over native FGF21, likely due to avidity effects from the dimerization of Fc (Fig. 5E). For Fc-FGF21 alanine mutants, reduced affinity for β-Klotho mostly resulted from an increase in rate of dissociation (Supplementary Fig. S5), suggesting that replacement by alanine at these sites may have destabilized the FGF21/β-Klotho complex. On the other hand, for arginine mutants, a combination of reduced association and increased dissociation contributed to attenuated affinity for β-Klotho (Supplementary Fig. S5), indicating that introduction of positive charges at these sites was incompatible with complex formation. Overall, there was a strong correlation between K_D_ determined by bio-layer interferometry and EC_50_ values obtained with the solid-phase binding assay (Fig. 5F and G).

**Figure 6:**
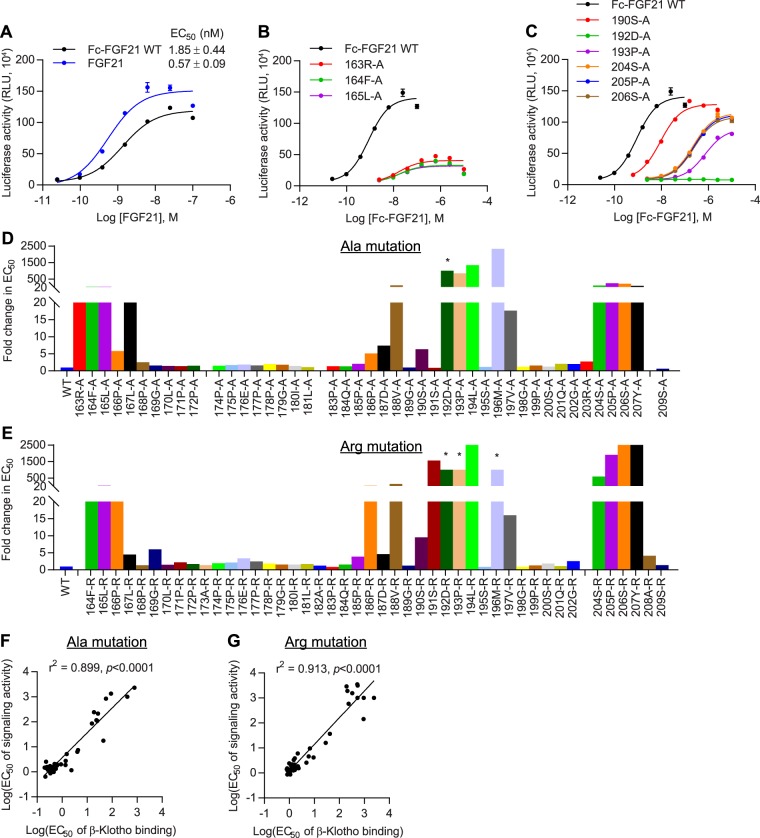
Interaction with β-Klotho determines signaling activity of FGF21. (**A**–**C**) Stimulation of Elk1-luciferase reporter activity by (**A**) FGF21 and WT Fc-FGF21 proteins, (**B**) Fc-FGF21 proteins containing mutations in the distal FGF21 trefoil core, and (**C**) Fc-FGF21 proteins containing mutations in the distal C-terminal tail in CHO reporter cells stably expressing human β-Klotho and FGFR1c. The curves are representative of three independent experiments performed in duplicates. (**D**,**E**) EC_50_ values determined from the activity assay described above for Fc-FGF21 (**D**) alanine and (**E**) arginine mutants. EC_50_ values are expressed as fold change relative to WT protein. Gaps were inserted to align the two graphs. *Curve did not converge. (**F**,**G**) Linear regression analysis of EC_50_ of β-Klotho binding versus EC_50_ of signaling activity for Fc-FGF21 (**F**) alanine and (**G**) arginine mutants.

To assess the functional impact of single residue mutations on FGF21 activity, we measured the ability of the mutants to activate β-Klotho/FGFR1c signaling in a cell-based reporter assay. Human β-Klotho and FGFR1c were stably transfected into CHO cells expressing luciferase reporter constructs^34,47^. As shown in Fig. 6A, despite possessing a stronger affinity for β-Klotho (Fig. 5E), WT Fc-FGF21 showed slightly lower potency and maximum response compared to native FGF21 in the activity assay. Of the mutant Fc-FGF21 proteins, replacement by alanine or arginine in three regions of the C-terminal tail resulted in reduced potency in the reporter assay (Fig. 6D and E). Two of the regions were those that showed positive peaks in HDX, mutations in which led to reduced affinity for β-Klotho. Regression analysis of the results of the biochemical versus cell-based assay affirmed that co-receptor affinity of the Fc-FGF21 mutants in these two regions correlated significantly with their signaling potency (Fig. 6F and G). Accordingly, in line with previous work^38,40^, this data supports the notion that β-Klotho binding to the C-terminal tail of FGF21 is a critical determinant of FGF21 functional activity.

The third region, from residue 163 to 167, lies in the putative β-trefoil core of FGF21 and constitutes the β12 strand. Single amino acid substitutions to this region resulted in a significant decrease in maximum response with a moderate effect on signaling potency in the reporter assay (Fig. 6B). Notably, affinity of these mutants for β-Klotho is retained (Fig. 5C and D), suggesting that this region does not interact directly with β-Klotho. Nevertheless, this region contributes to the overall conformation of FGF21 and, consequently, mutations may affect FGFR interaction, thereby dampening the degree of signal transduction that can be achieved. In contrast, mutations in the more distal region of the C-terminal tail did not significantly attenuate maximum receptor activation despite a reduction in potency (Fig. 6C), which suggests that while these mutants are weak agonists, they retain the ability to fully activate FGFR1c signaling.

### Identification of β-Klotho binding sites on FGF19

**Figure 7:**
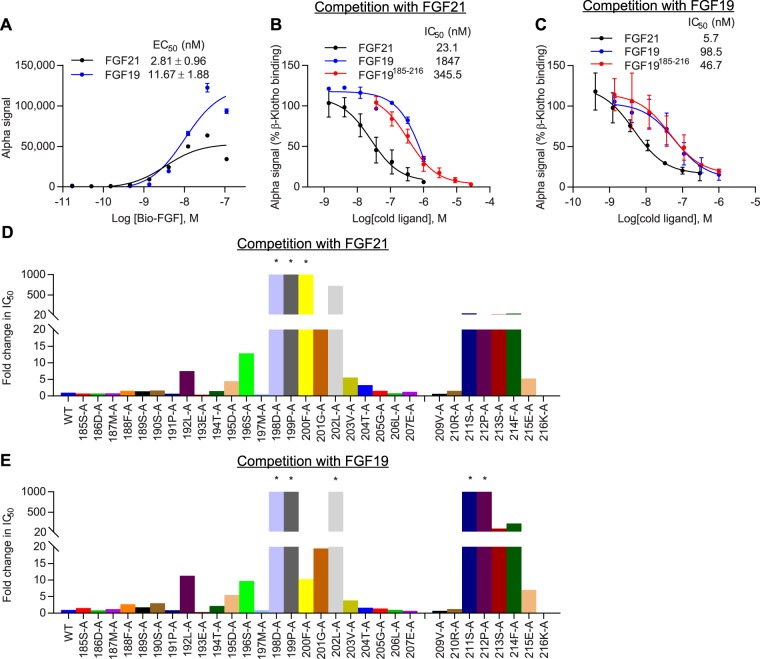
Two distinct, conserved sites in C-terminal regions of FGF19 and FGF21 mediate interaction with β-Klotho. (**A**) Binding of human FGF proteins to soluble human β-Klotho measured by AlphaScreen. (**B**,**C**) Inhibition of β-Klotho binding to (**B**) FGF21 and (**C**) FGF19 in the same AlphaScreen. The curves represent mean ± SD of three independent experiments performed in triplicates. EC_50_ and IC_50_ values represent mean ± SD of three independent experiments. (**D**,**E**) IC_50_ values against (**D**) FGF21 and (**E**) FGF19 determined from the AlphaScreen assay described above for FGF19 C-terminal mutant peptides. IC_50_ values are expressed as fold change relative to WT peptide. *Curve did not converge.

To identify β-Klotho-interaction sites on FGF19, we generated C-terminal FGF19 peptides and performed alanine scanning mutagenesis. Using AlphaScreen technology, the affinity of peptides for β-Klotho was assessed by their ability to compete with WT full-length FGF proteins (Fig. 7A–C). As shown in Supplementary Fig. S6, the relative affinity of FGF21 mutant peptides for β-Klotho binding correlated with that of full-length mutant proteins, validating the use of C-terminal peptides to determine co-receptor binding and supporting the C-terminus of FGF21 as the predominant domain that interacts with β-Klotho.

Similar to FGF21, mutations to the two highly conserved regions in the C-terminus of FGF19 interfered with the ability of the peptides to inhibit binding of full-length proteins to β-Klotho (Fig. 7D and E). In particular, single amino acid substitutions at 198D, 199P, 200F, 202L and the four residues near the distal C-terminus resulted in greater than 100-fold increase in IC_50_ compared to WT FGF19^185–216^, suggesting that these side chains interact directly with β-Klotho or are involved in stabilizing the C-terminal tail of FGF19. As in the case of FGF21, these results indicate that FGF19/β-Klotho complex formation likely involves concomitant occupancy of both regions on FGF19 C-terminus, as single amino acid mutations in either region were sufficient to abolish β-Klotho binding. Comparing the relative potency of mutant C-terminal FGF19 peptides and Fc-FGF21 mutants in β-Klotho binding showed very similar patterns of change among sequence-aligned residues (Supplementary Fig. S7), suggesting that FGF19 and FGF21 share common β-Klotho binding interfaces, which are consistent with previous findings^42^. However, differences between FGF19 and FGF21 with respect to co-receptor interactions may exist as a few residues exhibited differential changes when substituted by alanine. These are predominantly located in the first β-Klotho-binding site that interacts with KL1^14^. These differences may contribute to the differential affinity of FGF19 versus FGF21 for β-Klotho (Fig. 7B and C). Interestingly, although FGF19 and FGF21 exhibit interchangeable physiological effects *in vivo*^22,49,50^, previous *in vitro* work showed that in contrast to FGF21, FGF19 could interact with α-Klotho and a chimeric protein consisting of the KL1 domain from α-Klotho and the KL2 domain from β-Klotho (α/β-Klotho)^39^. Therefore, while both FGF19 and FGF21 adopt a two-binding site model and share overlapping binding sites on β-Klotho, the sequence differences between them may allow for more promiscuous binding by FGF19 to both α- and β-Klotho, but more specific binding by FGF21 to β-Klotho.

## Discussion

**Figure 8:**
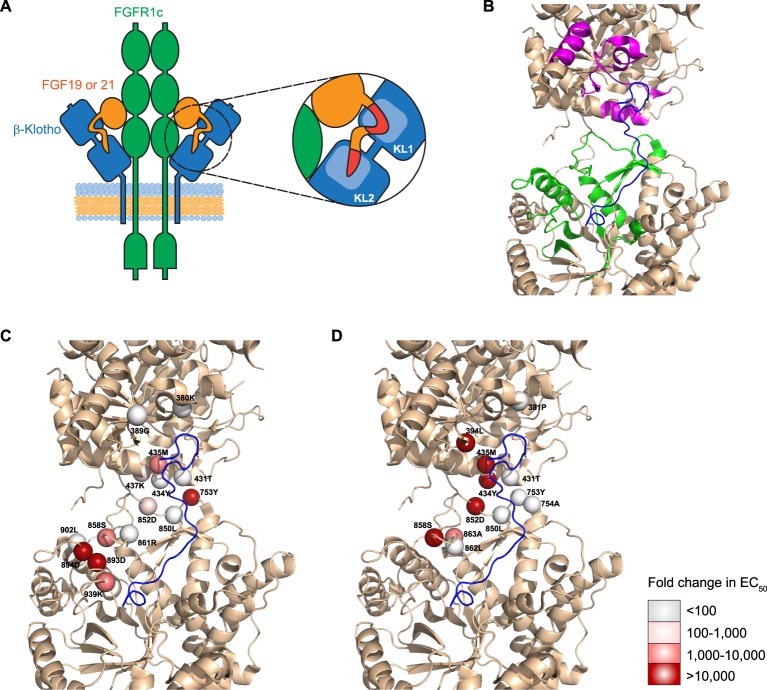
Proposed mechanism of FGF19 and FGF21 signaling. (**A**) Two highly conserved sites on the C-terminal tail of FGF19 and 21 engage with KL1 and KL2 of β-Klotho, respectively. Occupancy of both sites is required for activation of the β-Klotho/FGFR1c complex. The globular core domain of FGF19 and FGF21 may also interact with β-Klotho. (**B**) Mapping positive peaks identified by HDX-MS to the structure of ligand-bound β-Klotho ECD (PDB accession: 5VAQ). FGF21 C-terminus: blue. Positive peaks in KL1: magenta. Positive peaks in KL2: green. (**C**,**D**) Mapping potential FGF21-interacting residues identified by the solid-phase binding assay of (**C**) alanine and (**D**) arginine mutants to the structure of ligand-bound β-Klotho ECD (PDB accession: 5VAQ). Residues are represented as spheres with fold change in EC_50_ over WT denoted by different shades of red. FGF21 C-terminus: blue.

Presented herein, we mapped the interaction surface of FGF19 and FGF21 with their co-receptor, β-Klotho. We show that two highly conserved regions in the C-terminal tail of FGF19 and FGF21 interact with common binding sites on both the KL1 and KL2 domains of β-Klotho (Fig. 8A). While preparing this manuscript, two recent reports were published; one describing the crystal structure of the β-Klotho ECD bound to the C-terminal peptide of FGF21, and another describing the trimeric complex structure of α-Klotho ECD, FGFR1c ligand binding domain, and FGF23^14,15^. When we superimposed our KL1 structure and KL2 model on that of the published β-Klotho ECD (PDB accession 5VAN), we obtained overall C_α_ r.m.s.d. values of 0.29 Å and 1.12 Å, respectively, for KL1 and KL2, demonstrating virtually identical KL1 structures and a high degree of similarity in our KL2 model to the actual KL2 structure.

We mapped potential FGF21-interacting regions identified by our HDX-MS experiments onto the structure of the ligand-bound β-Klotho ECD (PDB accession 5VAQ). As shown in Fig. 8B, these regions occupy the inter-domain interface of β-Klotho and encompass a greater area than the actual interaction surface between β-Klotho and the FGF21 C-terminal peptide. Importantly, almost all the regions clustered around the two FGF21-binding sites on β-Klotho, indicating that enhanced protection observed in the presence of FGF21 was conferred by burial of the interface upon ligand binding. The only exception was a stretch of amino acids from 316–328 that constitutes part of the α5 helix in KL1. Our mutagenesis and binding studies suggest that this region is not directly involved in ligand interaction, but because of its proximity to the binding interface, FGF21 binding may stabilize and make this region more protected in the HDX experiment. Nevertheless, our HDX-MS results suggest that ligand binding does not change the overall folding of β-Klotho, consistent with the structural data that showed no change to the structure of either domain upon binding of FGF21 C-terminal tail to β-Klotho^14^.

For the KL1 domain, our HDX-MS experiments identified the β5-α6 loop, β7 strand, β7-α7 loop and α7 helix as part of the FGF21 interaction surface, which are highly consistent with the published structural data. For the KL2 domain, HDX-MS identified the β9 strand, β11 strand, β11-α11 loop, β12-α12 loop and the membrane proximal region of the KL2 domain as potential FGF21 interaction sites. Although our homology model of the KL2 domain adopts an overall TIM barrel fold typical of glycoside hydrolases that is very similar to the crystal structure, several loops were different, resulting in selection of some residues that were not solvent accessible for mutagenesis. Nevertheless, results of our binding and signaling assays are in keeping with the structural data. Key residues likely involved in stabilizing the FGF21/β-Klotho complex are highlighted as spheres in Fig. 8C and D. Their effects on ligand binding determined by the solid-phase binding assay are indicated by different shades of the spheres. Notably, residues 858S, 862L and 863A, located on the β14-α14 loop, and residues 893D, 894D and 902L, located on the β15-α15 loop, are on the upper rim of the ligand-interacting groove in the crystal structure, mutations to which may affect FGF21 access. Residues 434Y, 437K, 753Y, 850L and 852D do not make direct contact with FGF21. Rather, they are at the interface between KL1 and KL2, and may contribute to the overall configuration of the ligand binding pocket. These results align with the notion that ligand-binding regions from both KL domains act together to mediate interaction with FGF21, and are supported by a previous study using α-Klotho/β-Klotho chimeric proteins showing that only intact β-Klotho was capable of mediating FGF21 signaling^39^.

Consistent with previous reports^38–40^, our results indicate that the C-terminal domain of FGF21 is involved in co-receptor interaction. In these previous reports, serial truncations of the distal C-terminal tail were shown to progressively attenuate β-Klotho binding and FGF21 signaling activity^38,40^. Similarly, proteolytic cleavage of FGF21 C-terminus by fibroblast activation protein has been shown to render it completely inactive *in vivo*^51–53^. In this study, we narrowed down the interaction site to a region in the distal C-terminal tail spanning amino acids 204–208. Mutations to 204S, 206S and 207Y disrupted β-Klotho binding and attenuated FGF21 signaling activity; which is in agreement with the structural data^14^. Immediately before the distal C-terminus, the region spanning residues 198–202 does not make substantial contact with β-Klotho^14^ and mutations in this region did not affect co-receptor binding or FGF21 signaling.

While previous published work focused on the distal C-terminal tail^38,40^, we identified a second region, spanning amino acids 186–197 of FGF21, also critical for β-Klotho binding. This region has been shown to form a compact and rigid structure and is mostly composed of hydrophobic residues^14^. Importantly, the fact that single amino acid substitutions in either region were sufficient to abolish β-Klotho binding and FGF21 functional activity strongly indicate that both regions are necessary for co-receptor interaction. This finding is supported by previous results where, in *in vitro* signaling experiments and in animal models, removal of the distal binding region by truncation or proteolytic cleavage completely inactivated FGF21^38,40,52,53^. It is likely that interaction with β-Klotho at one region may be too weak to stabilize the FGF21/β-Klotho complex. Therefore, complex formation likely requires the concomitant occupancy of both regions in a cooperative manner for optimal binding geometry. In comparison, serial truncations of the N-terminus of FGF21 have been shown to have very little effect on the potency of FGF21 signaling but lead to a gradual reduction in maximum response^38,40^, arguing that the N-terminus plays a critical role in FGFR activation. These results suggest that regions upstream of the C-terminal tail of FGF21 are involved in FGFR interaction and signal activation. Therefore, while β-Klotho serves as the primary targeting receptor for FGF21, the weak, but detectable, binding of FGF21 to FGFR1c^34^ still contributes to the formation of the active ternary FGF21/β-Klotho/FGFR complex.

The data presented here demonstrates FGF19 engages β-Klotho in a manner highly similar to FGF21. Like FGF21, binding of FGF19 to β-Klotho occurs via two regions on its C-terminal tail. The amino acids in these two regions are highly conserved between FGF19 and FGF21, suggesting the presence of common co-receptor binding elements. This may explain, at the molecular level, the interchangeable effects of FGF19 and FGF21 on glucose and lipid homeostasis^22,49,50^. In line with this, in our AlphaScreen assays, both FGF19 and FGF21 could fully compete with each other for β-Klotho binding, albeit with different IC_50_ values. These results are consistent with previous findings that peptides derived from the C-terminal tail of FGF19 and FGF21 can inhibit each other for β-Klotho binding, and that chimeric proteins consisting of the core domain of FGF19 or FGF21 and the C-terminal tail of FGF21 or FGF19, respectively, bind β-Klotho with similar affinities as the native proteins^39,42^. Nevertheless, despite their similar physiological effects^22,49,50^, in contrast to FGF21, FGF19 could interact with α-Klotho and a chimeric protein consisting of the KL1 domain from α-Klotho and the KL2 domain from β-Klotho *in vitro*^39^. These differences may be attributed to primary sequence differences between FGF19 and FGF21 that allow for more promiscuous binding by FGF19 to both α- and β-Klotho, but more specific binding by FGF21 to β-Klotho. Under physiological conditions, differences in circulating concentrations, preference for FGFRs and expression patterns of Klotho proteins and FGFRs dictate the target tissues and primary functions of endocrine FGFs.

Collectively, the combined HDX-MS followed by site-directed mutagenesis approach described revealed two ligand-binding sites on β-Klotho, one on each KL domain, and two corresponding co-receptor binding sites on the C-terminal domain of FGF19 and FGF21. Notably, the use of biochemical and cell-based signaling assays in this approach enabled us to map the interaction surface, with single amino acid resolution, using full-length FGF21 protein. Additionally, these results are consistent with and support the recently published crystal structure of β-Klotho in complex with a C-terminal peptide of FGF21^14^. This suggests that interaction between the C-terminus and β-Klotho occurs independently of the core FGF domain. Furthermore, as structural information regarding the interaction of β-Klotho with FGF19 is lacking, we provide direct evidence showing that two segments on the C-terminal domain of FGF19 interact with the same two regions on the co-receptor engaged by FGF21. These two segments share a high degree of sequence similarity with corresponding co-receptor binding sites on FGF21, thus identifying common β-Klotho binding elements and unraveling the underlying molecular mechanism for the similar physiological effects of FGF19 and FGF21. During the review of our manuscript, a research article was published describing mapping of the β-Klotho interaction regions on FGF21 and FGF19^54^. These results confirm our findings on FGF19 and highlight the presence of conserved structural determinants that maintain co-receptor binding. Together, our work provides an evolved understanding of how endocrine FGFs interact with co-receptors for signal transduction, and could facilitate improved structure-guided design of FGF-related therapeutics for the treatment of metabolic disorders.

## Materials and Methods

### Hydrogen/Deuterium Exchange

Hydrogen/deuterium exchange experiments were performed as previously described^55^ on each target protein in free form and in a complex. Soluble human β-Klotho, and mature human FGF19 and FGF21 proteins were purified as previously described^34,38,47^. To form a protein complex, the proteins were mixed together with excess amount of binding partners (100% excess of FGFs when β-Klotho is the target and 50% excess of β-Klotho when FGFs are the targets), and buffer exchanged into PBS pH 7.0 using a 10K MWCO PES membrane (Vivaspin, Sartorius AG). The final concentration of each complex was 26 µM, assuming 100% recovery. Complex formation between β-Klotho and FGF was confirmed by size-exclusion chromatography (data not shown).

In each HDX experiment, the target protein and the complex were each diluted 5-fold into a phosphate/D_2_O buffer to initiate deuterium labeling. After incubation for a specific length of time at 25 °C, the labeling was quenched by a 4-fold dilution into a 0.45 M glycine buffer (pH 2.7, 1 °C) containing 0.625 M Tris (2-carboxyethyl) phosphine and 7.25 M urea. The quenched solution was then diluted 4-fold into a vial containing 0.2 mg/mL pepsin solution to initiate digestion. After a 6 minute digestion at 1 °C, the peptides were separated on a reversed-phase column followed by MS analysis. The entire labeling, quenching, digestion and injection process was performed on a LEAP HD-X PAL system controlled by HDxDirector (Leap Technologies). Peptides were separated on an Agilent 1290 Infinity system using a Waters CSH C-18 column at 1 °C (1.0 × 50 mm, 1.7 µ). The HPLC separation was performed at 1 °C with a 6-minute acetonitrile gradient of 1% to 40% (v/v) at a flow rate of 100 μL/min. Each mobile phase contained 0.04% (v/v) trifluoroacetic acid and 0.1% (v/v) formic acid. MS data were collected on a Thermo Scientific Orbitrap Fusion system at 120,000 resolution. Three peptides including bradykinin, angiotensin and Met-enkephaline (Sigma) were used as internal standards to control for run-to-run variations in deuterium back exchange. Tetrapeptide PPPI (synthesized by AnaSpec) was used as an internal standard to control for variation in intrinsic H/D exchange rate between the target protein and the complex. Negative control and fully deuterated control were performed for back exchange modeling. For peptide identification purpose, undeuterated target protein was also analyzed by the same procedure, except H_2_O was used instead of D_2_O in the labeling step, and five data-dependent low-resolution CID MS/MS were collected after each full scan.

The MS data were processed on MassAnalyzer (Biopharma Finder; Thermo Scientific) for fully automated feature extraction, peptide identification, deuterium uptake calculation, and HDX modeling to derive the average possible protection factor of each residue^55^. The protection factor is defined as the fold decrease in H/D exchange rate due to conformational protection, and the differential protection factor is defined as the difference in logP between the bound and free states, where P is the protection factor^55–58^.

### Site-directed mutagenesis

For site-directed mutagenesis of β-Klotho, the ECD of human β-Klotho (aa1–992) fused with a 6 × His tag at the C-terminus or the full-length human β-Klotho (aa1-1044; sequence accession number NM_175737) was cloned into the pTT14 expression vector and used as the PCR template. Solvent-accessible residues in the regions identified by HDX were individually mutated to alanine and arginine by site-directed mutagenesis PCR using the QuikChange II XL Site-Directed Mutagenesis Kit following the manufacturer’s protocol (Agilent). Primers were designed using the QuikChange Primer Design Program (Agilent). PCR product was digested with *DpnI* enzyme and transformed into XL-10 Gold competent *E*.*coli* cells. Transformed cells were selected on LB agar plates with 100 µg/mL ampicillin. Mutations were confirmed by DNA sequencing.

For site-directed mutagenesis of FGF21, DNA encoding the human mature FGF21 polypeptide (sequence accession number NM_019113) fused with the human Fc fragment at the N-terminus was cloned into the pTT5 expression vector as described previously^47^ and used as the PCR template. Residues in the C-terminus were individually mutated to alanine and arginine as described above for β-Klotho.

### Cell culture and transfection

β-Klotho and Fc-FGF21 mutant constructs were transiently transfected into CHO-EBNA1 (clone 3E7) and HEK 293-EBNA1 (clone 6E) cells, respectively, as previously described^59,60^. Briefly, CHO-EBNA1 cells were maintained in a chemically-defined CHO cell culture medium. HEK293-EBNA1 cells were maintained in Freestyle F17 medium (Invitrogen) supplemented with 6 mM GlutaMax (Invitrogen), 0.1% (w/v) poloxamer 188, and 25 µg/mL G418 (Corning). Cells were cultured in suspension format using shaker flasks on an orbital shaking platform rotating at 110 rpm in a humidified incubator with 5% CO_2_ at 37 °C. Cells were transfected during the exponential growth phase at 1 and 0.5 µg DNA/mL culture for CHO-EBNA1 and HEK293-EBNA1 cells, respectively, using linear PEI 25 kDa (Polysciences Inc.). Conditioned medium was harvested 6 and 7 days post-transfection for HEK293-EBNA1 and CHO-EBNA1 cells, respectively, by centrifuging cells at 4,000 rpm for 30 minutes and filtering the supernatant with a 0.2 µm PES membrane.

### Purification of Fc-FGF21 proteins

Fc-FGF21 mutants were purified from HEK293-EBNA1 cell culture medium by protein A affinity chromatography using MabSelect SuRe resin (GE Healthcare), eluted with 0.5% (v/v) acetic acid, pH 3.5, 150 mM NaCl and neutralized with 10% (v/v) of 1 M Tris-HCl, pH 8.0. Protein purity was confirmed by SDS-PAGE and immunoblotting.

### Quantification of β-Klotho concentration by ELISA

Nunc Maxisorp 96-well plates were coated overnight at 4 °C with 2 µg/mL of anti-β-Klotho antibody, 39F7, in PBS. Plates were washed twice with PBS plus 0.05% (v/v) Tween-20 (PBST) and blocked with 3% (w/v) BSA in PBS for 1.5 hours at room temperature. Conditioned media or His-tagged soluble recombinant β-Klotho protein were diluted in 1 × Reagent Diluent Concentrate 2 (R&D Systems) and incubated for 1.5 hours at room temperature. Plates were washed twice and incubated with biotinylated mouse anti-human β-Klotho antibody (R&D Systems) for 1 hour at room temperature. After 2 washes, plates were incubated with streptavidin-HRP (R&D Systems) for 30 minutes. Color development was performed with tetramethylbenzidine (TMB) substrate (BD Bioscience Pharmingen) followed by addition of 1 M H_2_SO_4_ as stop solution. Absorbance at 450 nm was read on a SpectraMax microplate reader (Molecular Devices).

### Solid-phase binding assay

Nunc Maxisorp 96-well plates were coated overnight at 4 °C with 2 µg/mL of a mouse anti-human β-Klotho antibody in PBS. Plates were washed twice with PBST and blocked with 3% (w/v) BSA in PBS for 1.5 hours at room temperature. After another wash, CHO-EBNA1 conditioned media containing 500 ng/mL of β-Klotho were diluted in 1 × Reagent Diluent Concentrate 2 (R&D Systems), added to the plates and incubated for 1.5 hours at room temperature. Plates were washed twice, incubated in PBST for 20 minutes and then washed another two times. FGF21 and an anti-β-Klotho antibody were biotinylated with EZ-Link Sulfo-NHS-LC-Biotin (Pierce) and added to the plates at the indicated concentrations. Plates were incubated for 1.5 hours at room temperature. After another wash, streptavidin-HRP (R&D Systems) was used for detection as described above. All assays were performed in duplicates. EC_50_ values were determined from a three-parameter logistic regression model with the least squares fit using GraphPad Prism version 7.

To assess binding of Fc-FGF21 mutants to β-Klotho, WT β-Klotho from CHO-EBNA1 conditioned media was captured as described above. After washing, purified Fc-FGF21 protein was added to the plates at indicated concentrations. Plates were incubated for 1.5 hours at room temperature, washed and incubated with 1 µg/mL mouse anti-human IgG Fc conjugated to HRP (Invitrogen) for another hour. After washing, TMB substrate was used for color development as described above.

### Luciferase reporter assay

To measure the signaling activity of β-Klotho mutants, ELK luciferase assay was performed in HEK293T cells that were stably transfected with reporter constructs containing 5 × UAS upstream of luciferase and Gal4 DNA-binding domain fused to ELK1. Briefly, HEK293T reporter cells were maintained in DMEM supplemented with 10% (v/v) FBS (Gibco) and 2 mM GlutaMax (Gibco) in a 37 °C incubator with 5% CO_2_. Cells were plated on 96-well plates in growth medium and incubated overnight. The next day, cells were transfected with expression vectors encoding full-length β-Klotho WT or mutants using the Lipofectamine 2000 transfection reagent (Invitrogen) according to the manufacturer’s protocol. Cells were serum-starved in DMEM supplemented with 5% (v/v) FBS (Gibco) and 2 mM GlutaMax (Gibco) overnight the day after transfection and treated with FGF21 for 6 hours at 37 °C with 5% CO_2_, after which the cells were lysed in Bright-Glo luciferase reagent (Promega). Luciferase activity was measured in relative luminescence units on the EnVision Multilabel Plate Reader (PerkinElmer) according to the manufacturer’s instructions. All assays were performed in duplicates. EC_50_ values were determined from a three-parameter logistic regression model with the least squares fit using GraphPad Prism version 7.

To measure the signaling activity of Fc-FGF21 mutants, ELK luciferase assay was performed in CHO cells stably transfected with human FGFR1c, β-Klotho and the two reporter constructs described above. CHO reporter cells were maintained in DMEM supplemented with 10% (v/v) dialyzed FBS (GE Healthcare Life Sciences), 6 µg/mL puromycin (Sigma) and 400 μg/mL hygromycin (Invitrogen) in a 37 °C incubator with 5% CO_2_. Cells were plated on 96-well plates in growth medium and incubated overnight. The next day, cells were serum-starved in Ham’s F-12K Nutrient Mixture (Corning) supplemented with 1% (w/v) BSA (Sigma) overnight. Following starvation, Fc-FGF21 protein was added to cells in starvation medium. Plates were incubated for 4 hours at 37 °C with 5% CO_2_, after which the cells were lysed in Steady-Glo luciferase reagent (Promega). Luciferase activity was measured and analyzed as described above.

### Bio-layer interferometry

Binding of β-Klotho to Fc-FGF21 was assessed by bio-layer interferometry on an Octet RED instrument (ForteBio). Briefly, β-Klotho ECD 6 × His was biotinylated with EZ-Link Sulfo-NHS-LC-Biotin (Pierce). Biotinylated β-Klotho protein at 10 µg/mL in kinetics buffer was loaded onto streptavidin biosensors (ForteBio). After an initial baseline step for 2 minutes in kinetics buffer, Fc-FGF21 mutants were associated to the immobilized β-Klotho for 5 minutes, followed by a dissociation step for 5 minutes in kinetics buffer. Blank binding cycles containing no Fc-FGF21 were included to correct for baseline drift. Results were fitted to a 1:1 binding model and analyzed by ForteBio Data Analysis software version 7.1 (ForteBio).

### Competition binding assay

FGF19 and FGF21 C-terminal WT and mutant peptides were custom synthesized and purified (>95% purity) (LifeTein LLC, South Plainfield, NJ). Binding of FGF19 and FGF21 peptides to β-Klotho was assessed using an AlphaScreen® assay. Briefly, 20 nM of β-Klotho ECD 6 × His, varying amounts of FGF19 and 21 peptides, and 40 nM of biotinylated human FGF19 or FGF21 protein were prepared in AlphaLISA universal buffer (PerkinElmer) and added to individual wells in 384-well white opaque plates (Greiner Bio-One). Subsequently, streptavidin donor beads and nickel chelate (Ni-NTA) acceptor beads (AlphaScreen Histidine detection kit; PerkinElmer) were diluted to 40 µg/mL and added to the plates. Total reaction volume was 8 µL. Plates were incubated for 3 hours at room temperature protected from light and read on the EnVision Multilabel Plate Reader (PerkinElmer) according to the manufacturer’s instructions. Assays were performed in triplicate. IC_50_ values were determined from a three-parameter logistic regression model with the least squares fit using GraphPad Prism version 7.

### Quantitative RT-PCR

RNA was extracted from cell pellets using the RNeasy Mini QIAcube Kit (Qiagen). After DNase (Promega) treatment to remove contaminating genomic DNA, 50 ng of RNA was used as template for quantitative real-time polymerase chain reactions (RT-PCR) performed on QuantStudio 7 Flex Real-Time PCR System (Thermo Fisher). Real-time monitoring of the following genes was performed using TaqMan fluorescent probes according to manufacturer’s protocol (TaqMan RNA-to Ct 1-Step Kit; Thermo Fisher): FGFR1 (Hs00915142_m1), FGFR2 (Hs01552918_m1), FGFR3 (Hs00179829_m1), FGFR4 (Hs01106908_m1), β-Klotho (Hs00545621_m1) and α-Klotho (Hs00934627_m1). The 18 S ribosomal RNA (Hs99999901_s1) was used as a housekeeping gene.

### Structure modeling

A homology model of KL2 was generated using the program Molecular Operating Environment (MOE) (Chemical Computing Group) with the crystal structure of KL1 domain^46^ as a template. We defined surface residues as residues with a solvent-accessible surface area larger than 10 Å^2^ and selected all surface residues on KL1 structure and KL2 model in PyMOL Molecular Graphics System, version 1.7 (Schrödinger). All structural figures were prepared using PyMOL.

### Statistical analysis

Data are presented as mean ± standard deviation (S.D.). Values were analyzed using GraphPad Prism version 7 (GraphPad Softwares Inc.).

### Data availability

The datasets generated during and/or analyzed during the current study are available from the corresponding author on reasonable request.

## Electronic supplementary material

Supplementary Figures
