# Supplementary material for: A systematic dissection of sequence elements determining β-Klotho and FGF interaction and signaling
Source: Sci Rep. 2018 Jul 23;8:11045. doi: 10.1038/s41598-018-29396-5 (PMC6056499; doi:10.1038/s41598-018-29396-5)
Supplement: Supplementary file 1 — Supplementary Figures [file 41598_2018_29396_MOESM1_ESM.pdf]

A systematic dissection of sequence elements determining β-Klotho and FGF interaction and signaling

Sally Yu Shi, Ya-Wen Lu, Jason Richardson, Xiaoshan Min, Jennifer Weiszmann, William G. Richards, Zhulun Wang, Zhongqi Zhang, Jun Zhang, and Yang Li

Supplementary Figure S1

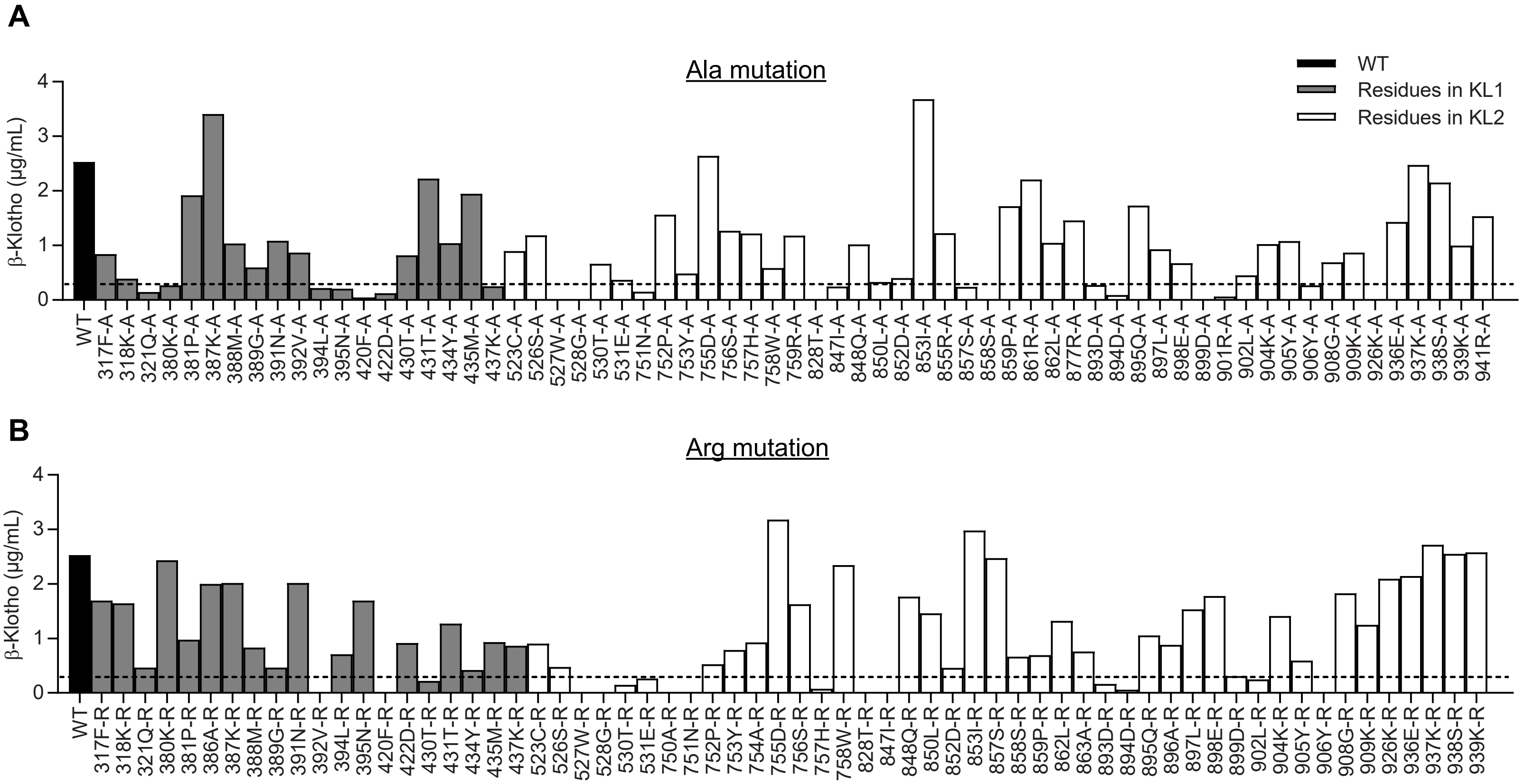

Supplementary Figure S1. Expression of β-Klotho (A) alanine and (B) arginine mutants in CM determined by ELISA. Dotted lines represent the cutoff point (0.3 µg/mL) for subsequent solid-phase binding assay.

Supplementary Figure S2

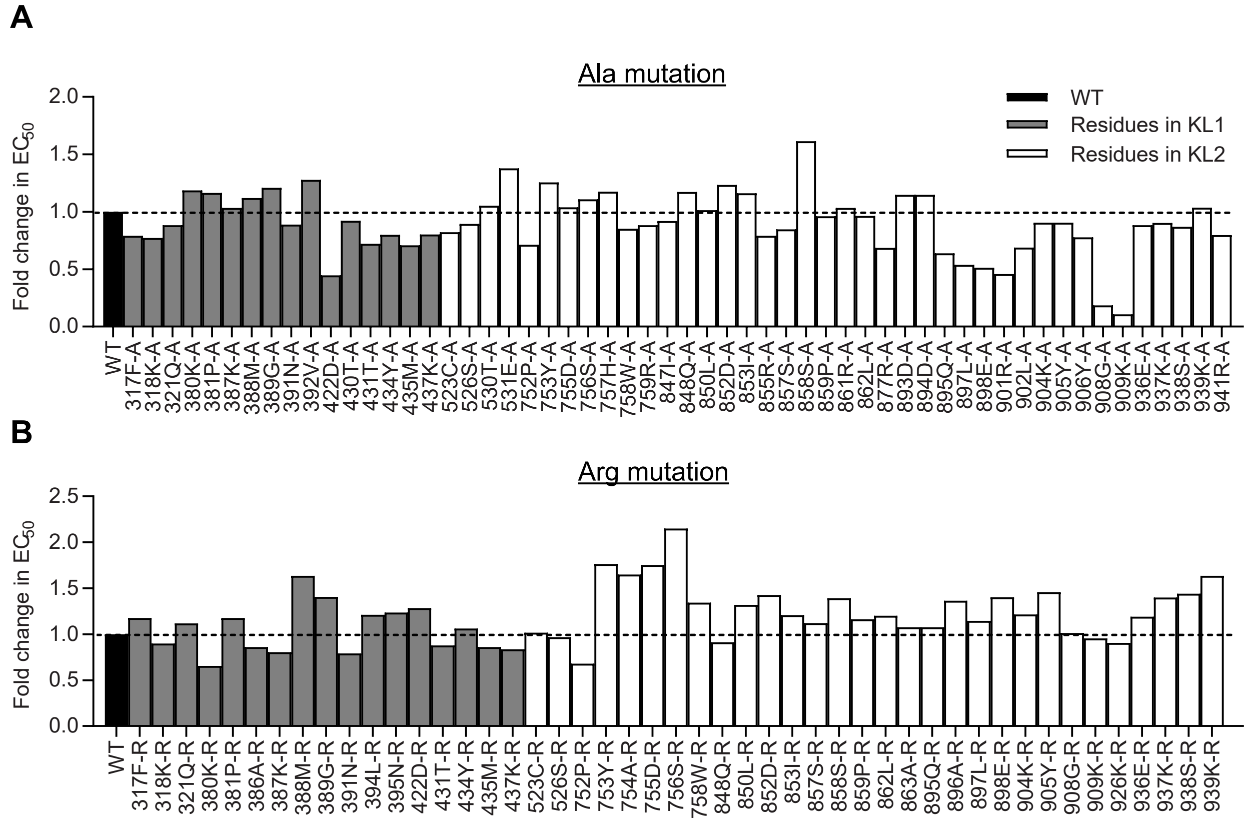

Supplementary Figure S2. EC_{50} values determined from the solid-phase binding assay between β-Klotho and an anti-β-Klotho antibody, 39F7, for (A) alanine and (B) arginine mutants. EC_{50} values are expressed as fold change relative to WT CM.

Supplementary Figure S3

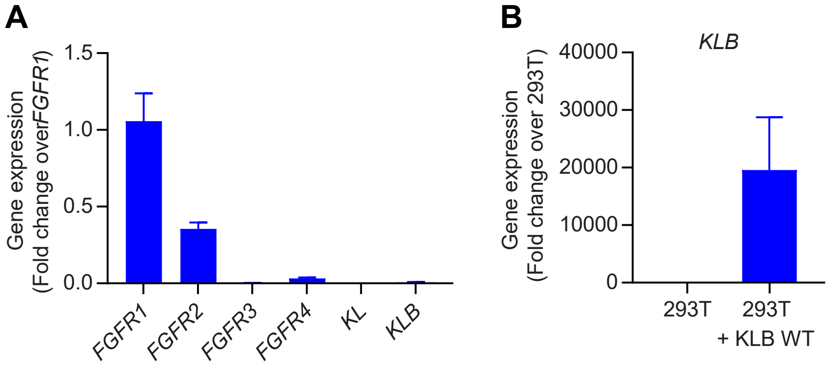

Supplementary Figure S3. (A) Expression of FGFR isoforms, α-Klotho and β-Klotho in 293T cells measured by quantitative RT-PCR. (B) Expression of β-Klotho in 293T cells transiently transfected with full-length WT β-Klotho construct measured by quantitative RT-PCR. Results are mean ± SD of two independent experiments.

Supplementary Figure S4

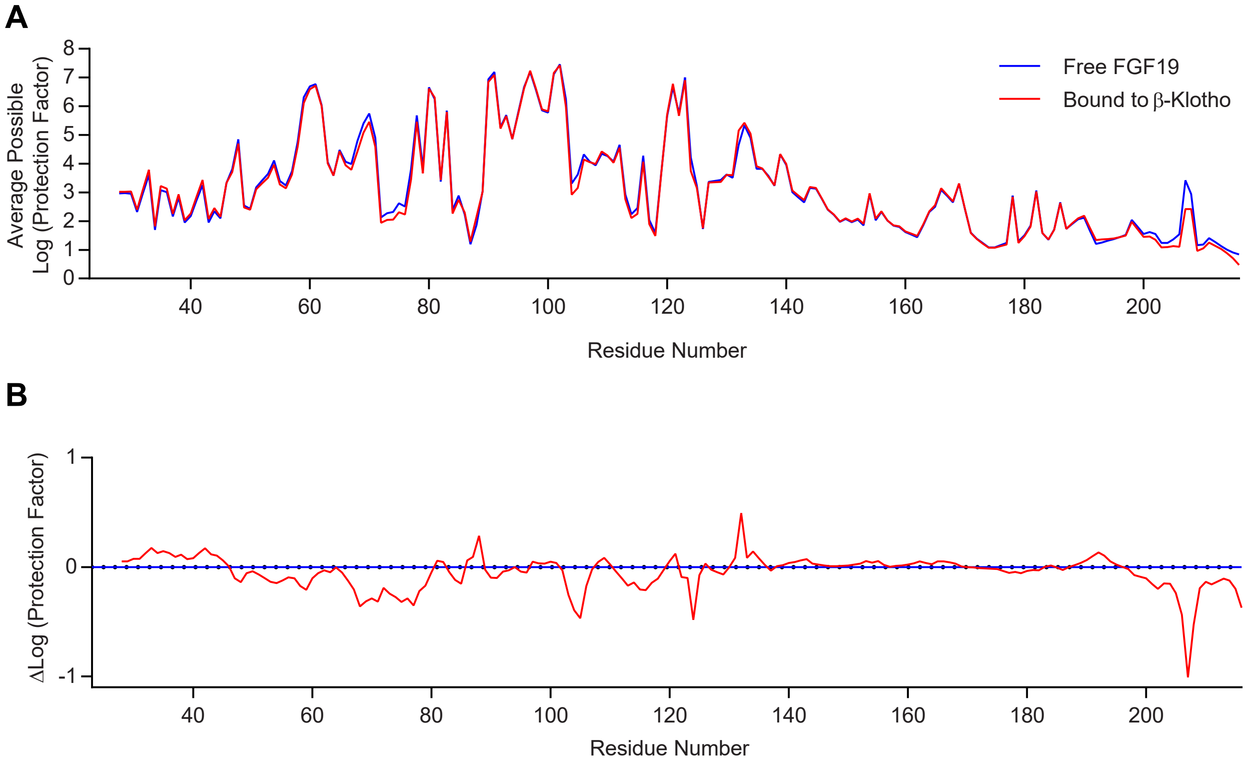

Supplementary Figure S4. Identification of potential β-Klotho interaction regions on FGF19 by HDX-MS. (A) Average possible protection factor plot and (B) differential protection factor plot of β-Klotho-bound and free FGF19.

Supplementary Figure S5

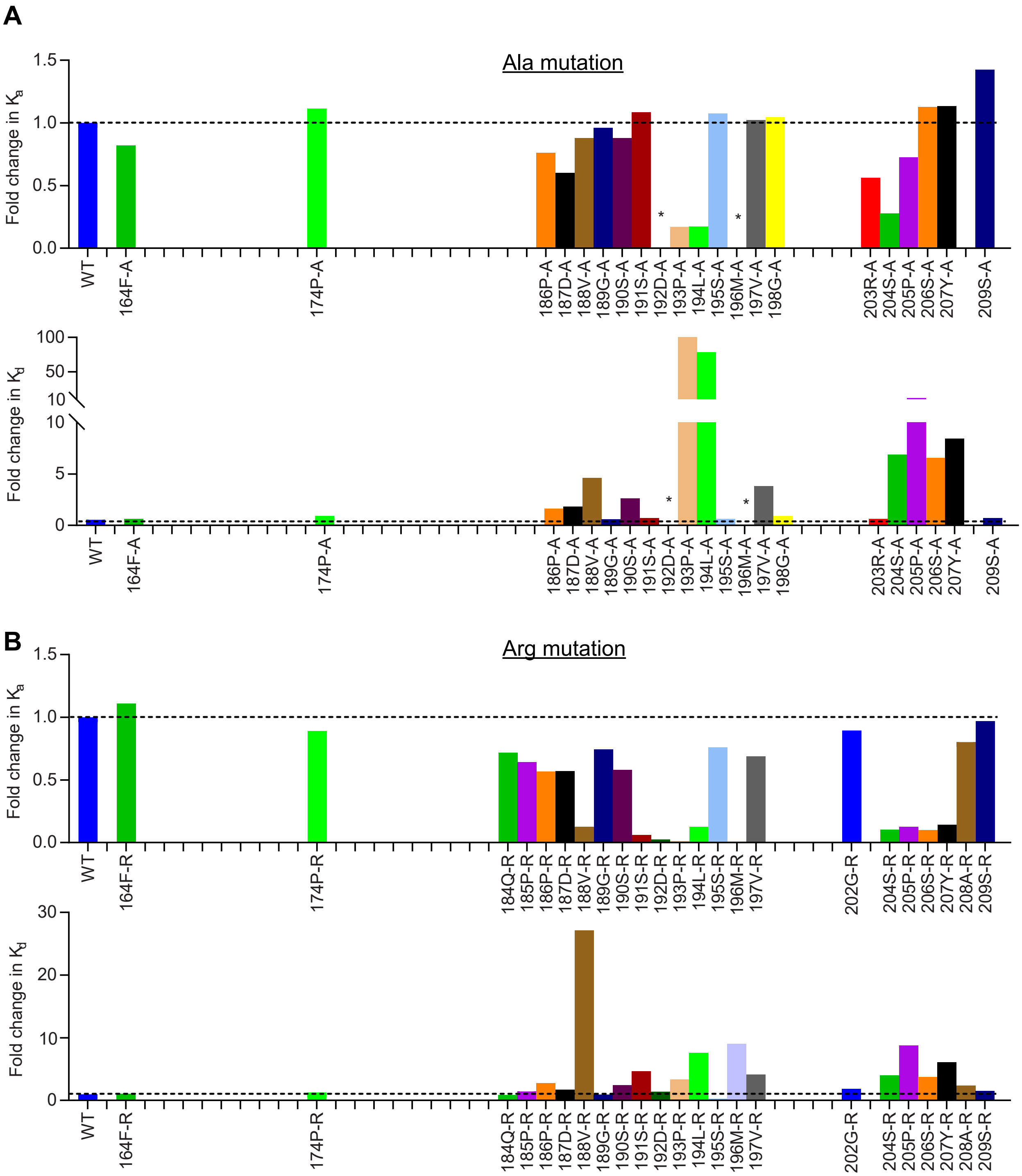

Supplementary Figure S5. Analysis of β-Klotho binding kinetics of selected Fc-FGF21 (A) alanine and (B) arginine mutants by bio-layer interferometry. Top, association rate constant (K_a); bottom, dissociation rate constant (K_d). Values are expressed as fold change relative to WT. *, could not be estimated.

Supplementary Figure S6

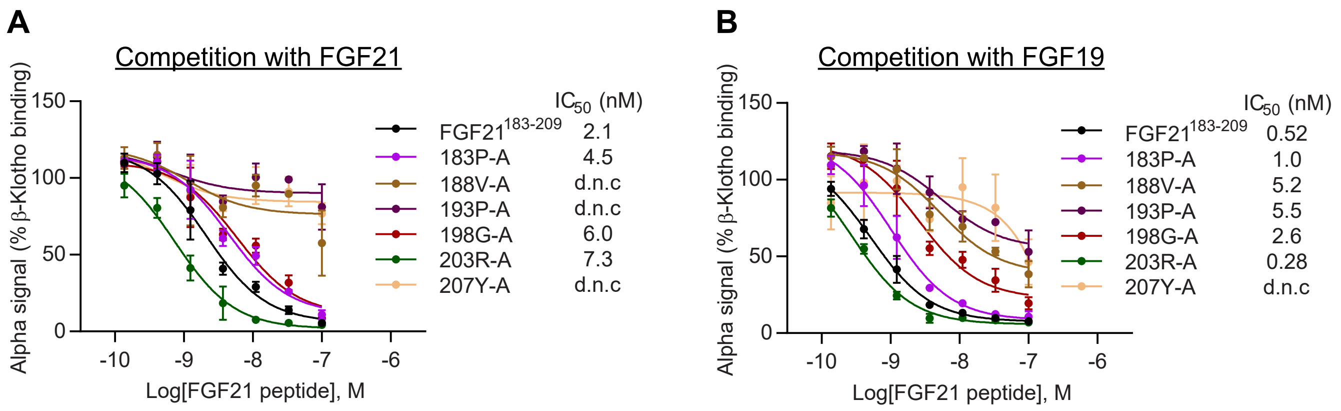

Supplementary Figure S6. Inhibition of β-Klotho binding to (A) FGF21 and (B) FGF19 by FGF21 C-terminal mutant peptides measured using AlphaScreen. d.n.c., did not converge.

Supplementary Figure S7

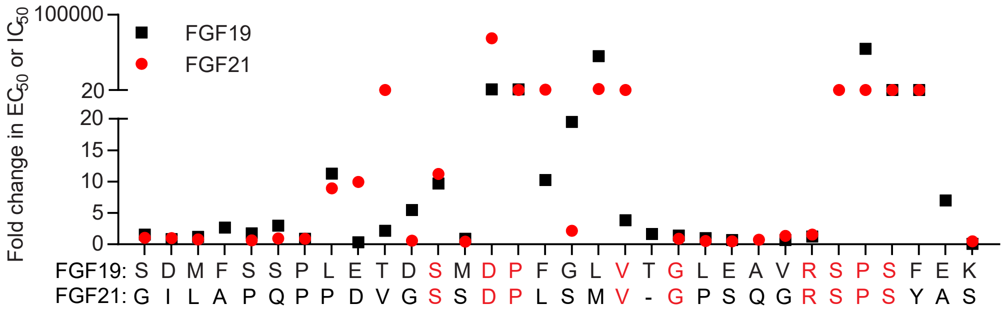

Supplementary Figure S7. IC_{50} of FGF19 mutant peptides and EC_{50} of Fc-FGF21 alanine mutants graphed along aligned C-terminal sequences. Conserved residues are colored in red.
